# Evaluation of the Effectiveness of Telehealth Chronic Disease Management System: Systematic Review and Meta-analysis

**DOI:** 10.2196/44256

**Published:** 2023-04-27

**Authors:** Ziyan Xiao, Xiuping Han

**Affiliations:** 1 Research Center Intelligent Equipment and Technology Yangtze Delta Region Institute of Tsinghua University Jiaxing, Zhejiang China; 2 Department of Statistics and Actuarial Science Faculty of Science The University of Hong Kong Hong Kong Hong Kong

**Keywords:** telehealth chronic management system, management system, effectiveness evaluation, quality of life, mental health, depression, anxiety, fatigue, self-management, self-efficacy, medical adherence, disease management, chronic condition, chronic disease, telemedicine, meta analyses, meta analysis, systematic, review method, search strategy, literature search

## Abstract

**Background:**

Long-term daily health monitoring and management play a more significant role in telehealth management systems nowadays, which require evaluation indicators to present patients’ general health conditions and become applicable to multiple chronic diseases.

**Objective:**

This study aims to evaluate the effectiveness of subjective indicators of telehealth chronic disease management system (TCDMS).

**Methods:**

We selected Web of Science, ScienceDirect, Scopus, Cochrane library, IEEE, and Chinese National Knowledge Infrastructure and Wanfang, a Chinese medical database, and searched papers published from January 1, 2015, to July 1, 2022, regarding randomized controlled trials on the effectiveness of the telehealth system on patients with chronic diseases. The narrative review summarized the questionnaire indicators presented in the selected studies. In the meta-analysis, Mean Difference (MD) and Standardized Mean Difference (SMD) with a 95% CI were pooled depending on whether the measurements were the same. Subgroup analysis was conducted if the heterogeneity was significant, and the number of studies was sufficient.

**Results:**

Twenty RCTs with 4153 patients were included in the qualitative review. Seventeen different questionnaire-based outcomes were found, within which quality of life, psychological well-being (including depression, anxiety, and fatigue), self-management, self-efficacy, and medical adherence were most frequently used. Ten RCTs with 2095 patients remained in meta-analysis. Compared to usual care, telehealth system can significantly improve the quality of life (SMD 0.44; 95% CI 0.16-0.73; *P*=.002), whereas no significant effects were found on depression (SMD −0.25; 95% CI −0.72 to 0.23; *P*=.30), anxiety (SMD −0.10; 95% CI −0.27 to 0.07; *P*=.71), fatigue (SMD −0.36; 95% CI −1.06 to 0.34; *P*<.001), and self-care (SMD 0.77; 95% CI −0.28-1.81; *P*<.001). In the subdomains of quality of life, telehealth statistically significantly improved physical functioning (SMD 0.15; 95% CI 0.02 to 0.29; *P*=.03), mental functioning (SMD 0.37; 95% CI 0.13-0.60; *P*=.002), and social functioning (SMD 0.64; 95% CI 0.00-1.29; *P*=.05), while there was no difference on cognitive functioning (MD 8.31; 95% CI −7.33 to 23.95; *P*=.30) and role functioning (MD 5.30; 95% CI −7.80 to 18.39; *P*=.43).

**Conclusions:**

TCDMS positively affected patients’ physical, mental, and social quality of life across multiple chronic diseases. However, no significant difference was found in depression, anxiety, fatigue, and self-care. Subjective questionnaires had the potential ability to evaluate the effectiveness of long-term telehealth monitoring and management. However, further well-designed experiments are warranted to validate TCDMS’s effects on subjective outcomes, especially when tested among different chronically ill groups.

## Introduction

Chronic disease has long been a significant concern in the health management industry. According to Global Burden of Disease 2015 Studies, chronic diseases accounted for more than 50% of global deaths, among which cardiovascular diseases, diabetes, and Chronic Obstructive Pulmonary Disease (COPD) were the 3 main sources [1]. In China, 75.8% of older adults older than 60 years had at least 1 type of chronic disease [2], and in Europe, 70% of health care expenses were spent on chronic disease management [3]. With the aging population in many countries, these figures are projected to rise in the following years, resulting in severe social burdens. Therefore, it is necessary to develop chronic disease management systems. Efficiency and effectiveness are 2 major concerns.

In recent years, telehealth has been rapidly developed to realize more efficient chronic health management. By definition, telehealth is a way to provide health care remotely facilitated by mobile technology like smartphone apps and the internet [4]. Compared with community care without digitalized tools, telehealth can significantly reduce the time and costs spent on health status monitoring [5]. There are also fewer requirements on labor and site, as care leaders and offline participation were no longer indispensable [6]. Additional benefits include decreased hospitalization rates [7] and decreased manual errors in entering data, since mobile devices can record medical data frequently and remotely [8]. During the COVID-19 pandemic, telehealth also helped to alleviate patients’ depression and anxiety by providing remote treatment and consultation channels when offline hospital visits were impeded [9]. It seems that telehealth was not just an efficient system but also effective. From a study on patients’ perception regarding telemonitoring, nearly 90% of patients felt satisfied with the care and became more knowledgeable about their disease [10].

However, there are some challenges in measuring the effectiveness of Telehealth Chronic Disease Management System (hereinafter referred to as TCDMS). Nowadays, TCDMS tends to expand services from addressing hospital-based, acute conditions to managing the chronic disease at home [4], focusing more on long-term general health monitoring. This trend fits with the natural needs of the vulnerable elderly [11] and, in the meantime, requires stable and precise indicators that can be applied to various chronic diseases and complications. Nevertheless, past studies used to restrict to a single disease and evaluate the effectiveness of intervention using corresponding clinical outcomes. These disease-specific indexes may fail to attain broad applicability and reflect the general health condition in long-time daily monitoring.

Subjective outcomes, however, can be possible candidate indicators. Some typical indexes are Quality of Life (QoL), psychological well-being, self-management, and medical adherence. These questionnaires can be applied to different diseases and present some important aspects of patients’ conditions, including physical and mental well-being. Some previous studies also adopted subjective measurements as the main outcomes. In 2013, Cartwright et al [12] conducted a large-scale Randomized Controlled Trial (RCT) on the effects of telehealth systems, using QoL and mental well-being as the main results indicators. Some recent reviews also reported the positive effects of telehealth on some subjective outcomes, such as those on mental health and QoL of patients with breast cancer and adherence and hospital admission of patients with cardiovascular conditions [13,14]. However, as far as we know, there is a lack of research on whether subjective indicators could be effective indicators of long-term health monitoring and management across multiple chronic diseases.

Therefore, this study hopes to obtain evidence for a thorough evaluation of the TCDMS. This study was to conduct a systematic review and meta-analysis to summarize the related research based on subjective indicators among chronically ill patients. The main objectives include: (1) investigating the current research conditions of adopting questionnaire-based subjective outcomes to evaluate the effectiveness of TCDMS and (2) synthesizing the effects of TCDMS on subjective outcomes and analyzing the heterogeneity of studies results.

## Methods

### Literature Search

The literature in this paper was searched in the English databases Web of science, ScienceDirect, Scopus, Cochrane library (including Embase, PubMed, ICTRP, CT.gov, and CINAHL), IEEE, and Chinese databases Chinese National Knowledge Infrastructure and Wangfang. To focus on the recent advances, we restricted papers to those published during the period from January 1, 2015, to July 1, 2022. Language was restricted to English and Chinese. In recent 2 years, there have been many studies on telehealth in the context of COVID-19. Since the pandemic may influence the patient’s psychological states and experiment conditions, we decided to eliminate related papers to reduce the potential intervention and obtain more consistent results in the long run.

We used keyword combinations in the Title or Title/Abstract/Keywords fields. Keywords were selected and categorized into 4 categories: health technology (subject of the study), evaluation (objective of the study), RCT (study design), and excluded keywords, namely query #1 to #4, respectively. An additional query #5 was to set time limits from January 1, 2015, to July 1, 2022. The overall search strategy was #1 AND #2 AND #3 AND (NOT #4) AND #5. Table 1 presents the hierarchical search query and all keywords.

**Table 1:** Literature search strategy. The overall search query was #1 AND #2 AND #3 AND (NOT #4) AND #5.

Search	Keywords
#1 Title	(e-health OR m-health OR mobile health OR telehealth OR digital health OR remote health OR web-based, health OR internet-based, health)
#2 Title	(Evaluation OR Effects OR Results OR Assessment OR Influence)
#3 Title/Abstract/Keywords	(Randomized Controlled Trial)
#4 Title/Abstract/Keywords	(Covid-19 OR Protocol OR Design)
#5 Time range	January 1, 2015, to July 1, 2022

### Eligibility Criteria

Literature was included if it satisfied the following requirements:

1. Study design: RCT
2. Patients: patients older than 18 years with chronic disease, including diabetes, cardiovascular disease, previous cancer, and mental health issues.
3. Intervention: internet+health management system intervention
4. Control: receive usual care or in waitlist control
5. Outcomes: at least 1 of the following was quantitatively measured in the study: quality of life, depression, anxiety, and self-management.

Exclusion criteria were as follows:

1. Papers without full text;
2. Non-RCT studies, including protocol, secondary analysis of RCT results, scoping analysis, systematic review, and meta-analysis;
3. Intervention did not fulfill the requirements of the TCDMS, especially without remote monitoring and health management functions. For instance, a system with self-learning module only or interventions based on telephone or short messages, were regarded as ineligible studies;
4. Studies with insufficient outcome data;
5. Studies with ineligible patients or outcomes.

### Data Extraction and Quality Assessment

Data were extracted from the full text of each paper, including literature characteristics, patient characteristics, intervention description, outcomes, and corresponding measurement methods. The extracted information is listed in Multimedia Appendix 1 [15-35]. Quantitative outcome data were collected by 2 reviewers independently, cross-checked, and integrated into an Excel file (Microsoft Corp).

We used the Cochrane Risk of Bias Assessment Tool (version 2.0, RoB. 2.0) to assess the quality of included studies. Two reviewers independently reviewed the papers, and any disagreements were resolved by consulting with a third researcher.

### Statistical Analysis

This paper used Review Manager 5.4 (Cochrane) for statistical analysis. Mean Difference (MD) and Standardized Mean Difference (SMD) were used for continuous outcomes depending on whether different scales were adopted in measurement. Any individual studies with eligible and sufficient data would be used as a unit of meta-analysis. We also noted that some outcomes might have several common domains, and thus we would further integrate the subdomains that were presented in multiple studies. We used the outcome data presented in the published paper. We did not further contact researchers for eligible studies with missing data.

*I*^2^ statistics were used to measure the heterogeneity among studies. A fixed-effects model was used when *I*^2^<50%, and a random-effects model was used otherwise. We conducted subgroup analysis for outcomes with significant heterogeneity, that is, an *I*^2^ larger than 75%. Subgroups were divided based on measurement scales, sample size, the average age of patients, and the follow-up period.

Considering the limited number of studies included in the meta-analysis, we could not conduct meta-regression and use funnel plots to evaluate the potential publication bias.

## Results

### Search Results

**Figure 1:**
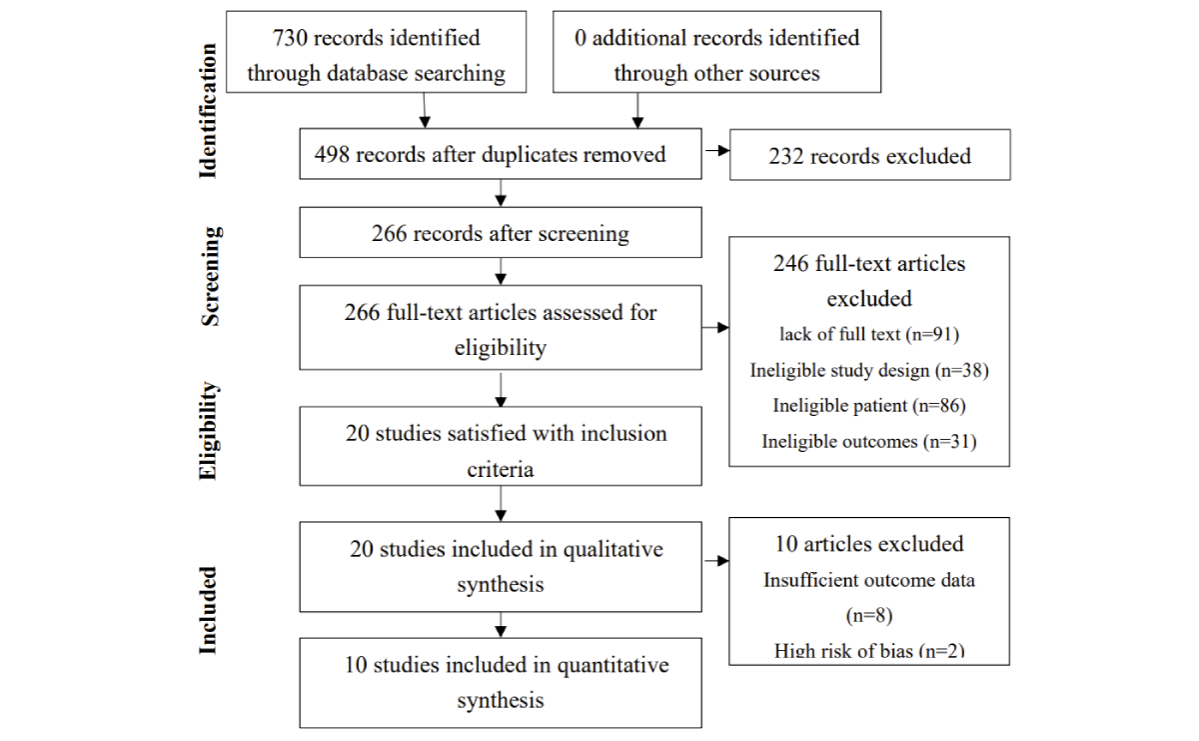
Preferred Reporting Items for Systematic Reviews and Meta-Analyses flow diagram of study selection.

Figure 1 illustrates the process of paper selection. Electronic database search yielded 730 studies, and no additional studies were included from other resources. After deduplication, there remained 498 studies. By screening the title and abstract, we excluded 232 ineligible studies and reviewed the full text of the rest 266 studies. Twenty studies satisfied all inclusion criteria, ready for the risk of bias assessment and qualitative synthesis. Finally, 10 studies with sufficient eligible outcome data were selected for quantitative analysis.

### Risk of Bias

**Figure 2:**
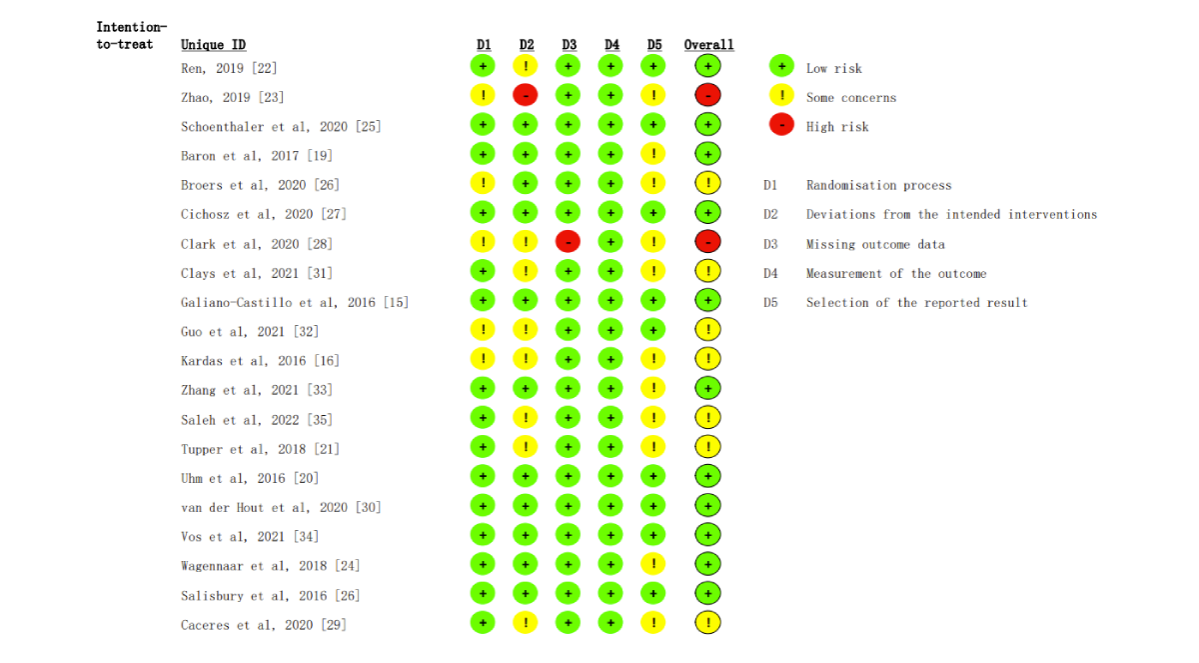
Risk of bias of the selected studies.

Risk of bias assessment of selected studies is illustrated in Figure 2. The major source of bias came from the potential deviations from intended interventions, as some of the researches did not use appropriate analysis, such as intention-to-treat, to assess the effects of assignments. Another source of bias existed in the selection of reported results since many of the research protocols were not available. Some studies claimed to be RCT, whereas they did not specify the blinding process in randomization. To conclude, 7 studies had a moderate level of risk of bias, and 2 studies with a high risk of bias were excluded from the quantitative analysis.

### Systematic Review

Demographic information of the included studies is shown in Multimedia Appendix 1. There were 20 studies and 4153 patients involved in the qualitative analysis, with an average age of 59.96. Sixteen (80%) studies had a mean patient age over 55. Male patients accounted for 54.82% of a test population among 18 studies with available gender data. There were 2 studies on breast cancer without gender ratio in patient demographics, but by default, they all referred to female patients [15,20]. Six studies focused on patients with cardiovascular diseases, 5 on patients with cancer or survivors, 5 on diabetes, 1 on COPD, 1 on depression, 1 on hypertension and 1 on patients with hypertension, or diabetes.

These studies were RCTs conducted in 11 countries across Europe, North America, and Asia. The follow-up period ranged from 4 weeks to 12 months. The intervention arm was a telehealth management system, which should at least consist of monitoring and management functions. The controlled measures included receiving usual care, being on the waitlist, and receiving health-related brochures. All questionnaire-based self-reported outcomes are presented in the last column of Multimedia Appendix 1.

Considering the availability of data, 10 studies were selected for the meta-analysis. There were 2095 patients in total, with an average age of 57.09. Male patients accounted for 51.5%, and in 2 studies, the number of male and female patients was unavailable. Among all outcome indicators, QoL, depression, anxiety, and self-care had more than 1 study to synthesize. Consequently, these indicators constituted the outcomes evaluated in the meta-analysis. The table of characteristics of included trials is attached in Multimedia Appendix 1.

We summarize the frequencies and measurements of outcomes in Table 2. Health-related quality of life was the most frequently used outcome among different chronic diseases. Some studies used general quality of life questionnaires, such as the Short Form (SF-36) Health Survey [36]. Other studies used disease-specific questionnaires, such as diabetes-specific and cancer-specific scales. Many QoL scales thoroughly covered different perspectives of patients’ health, including physical, mental, and social functioning.

Adherence, self-care, self-efficacy, depression, and anxiety are less frequently used outcomes; yet, they may have the potential of indicating health conditions among multiple chronic diseases. The 8-item Morisky Medication Adherence Scale questionnaire is used to evaluate the adherence level [37], and the Health Education Impact Questionnaire to evaluate the self-efficacy level [38]. There was no consensus on the measurement of self-care. Psychological well-being was another meaningful outcome, among which depression and anxiety were frequently measured, although the standard questionnaire to measure these 2 outcomes varied. A 10-item Center for the Epidemiological Studies of Depression Short Form [39], Patient Health Questionnaire-9 [40], and Beck Depression Inventory [41] could be used for measuring depression levels, while State-Trait Anxiety Inventory [42] and Generalized Anxiety Disorder Assessment [43] could be used for anxiety.

The remaining indicators were observed only once among all studies. Among these, pain, physical activity, distress, and fatigue were also separate indicators of QoL. Quality-adjusted life year was calculated using the EuroQol-5D index, which has a similar meaning to the quality of life. Lifestyle was also measured in multiple studies, whereas only 1 used a general questionnaire, and the others measured specific items such as physical activities, smoking, or drinking condition. Satisfaction and symptom burden can be found in articles that were not selected; yet in this review, there was a lack of studies containing these 2 outcomes. Health literacy, social support, and type D personality were rarely observed among studies.

**Table 2:** Frequencies and measurements of outcomes found in the selected studies.

Outcomes	Frequencies of appearances, n (%)	Measurements
Health-related quality of life	14 (70)	General questionnaire: EORTC QLQ-C30,^a^ EuroQoL 5D, SF-36,^b^ SF-12, WHOQOL,^c^ 15D questionnaire Disease-specific questionnaire: Atrial Fibrillation Effect on Quality-of-Life
Adherence	5 (25)	MMAS-8^d^ questionnaire, self-reported adherence condition, dose taken rate, and attendance rate
Self-care	5 (25)	Summary of diabetes self-care activities assessment, self-care of heart failure index European Heart Failure Self-care Behaviour [44]
Depression	4 (20)	CESD-10,^e^ PHQ-9,^f^ BDI^g^
Anxiety	4 (20)	STAI-6,^h^ STAI-Y,^i^ GAD-7^j^
Self-efficacy	3 (15)	HeiQ^k^
Pain	2 (10)	Brief Pain Inventory
Quality adjusted life year	2 (10)	EuroQoL 5D
Physical activity	2 (10)	International Physical Activity Questionnaire Short form, Global Physical Activity Questionnaire
Distress	1 (5)	Diabetes Distress Scale
Fatigue	1 (5)	Revised Piper Fatigue Scale
Health literacy	1 (5)	Short-Test of Functional Health Literacy in Adults
Lifestyle	1 (5)	Health Promotion Lifestyle Profile
Satisfaction	1 (5)	Client Satisfaction Questionnaire—8
Social support	1 (5)	Patient Reported Outcomes Measurement Information System
Symptom burden	1 (5)	Memorial Symptom Assessment Scale-Heart Failure
Type D personality	1 (5)	Type D scale

^a^EORTC QLQ-C30: European Organization for Research and Treatment of Cancer Core Quality of Life.

^b^SF-36: Short form (36) Health Survey.

^c^WHOQOL: A Quality of Life assessment developed by the World Health Organization.

^d^MMAS-8: Eight-item Morisky Medication Adherence Scale.

^e^CESD-10: 10-item Center for the Epidemiological Studies of Depression short form.

^f^PHQ-9: Patient Health Questionnaire-9.

^g^BDI: Beck Depression Inventory.

^h^STAI: State-Trait Anxiety Inventory.

^i^STAI-Y: State-Trait Anxiety Inventory, Form Y.

^k^GAD-7: Generalized Anxiety Disorder Assessment.

^k^HeiQ: Health Education Impact Questionnaire.

### Meta-analysis

#### Quality of Life and Separate Domains in Quality of Life

##### General Quality of Life

**Figure 3:**
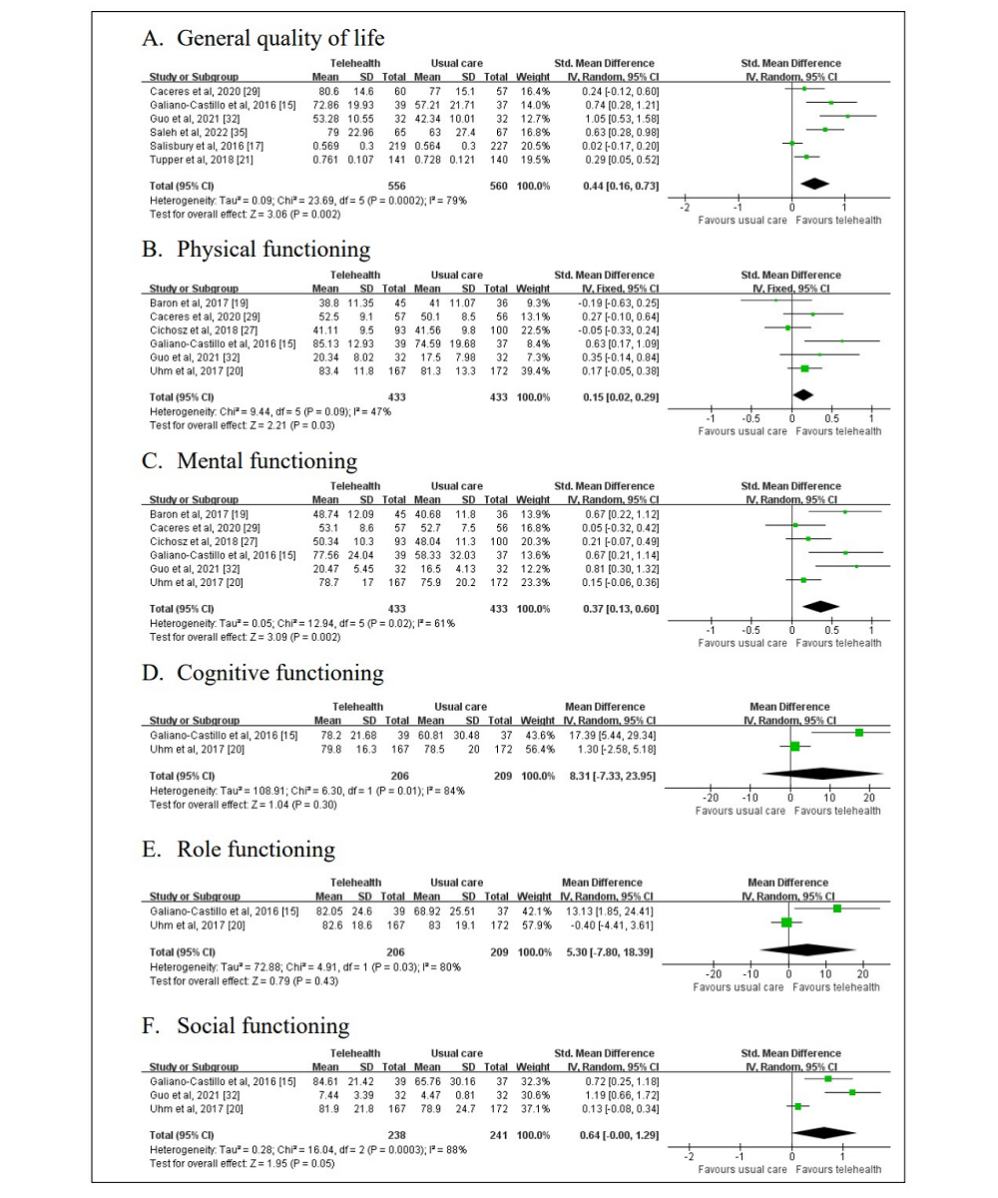
Forest plots of health-related quality of life and separate domains in quality of life: (A) General quality of life, (B) physical functioning, (C) mental functioning, (D) cognitive functioning, (E) role functioning, and (F) social functioning.

Six studies reported the general QoL score, with a total of 1116 patients involved (Figure 3A). SMD method was used considering different measurement scales. Significant heterogeneity across the studies could be observed (*P*=.0002, *I*^2^=79%), so a random effects model was used to calculate the mean effect size. The result was that the positive effect of telehealth intervention was statistically significant (SMD 0.44; 95% CI 0.16-0.73; *P*=.002).

To further explore the significant heterogeneity, a subgroup analysis was conducted on the measurement scales, average age of patients, sample size, and follow-up period, as shown in Table 3. It was found that grouping by measurement scales or average age of patients was not sufficient to resolve the heterogeneity, while telehealth tended to pose a stable positive influence on the older generation (SMD 0.50; 95% CI 0.20-0.81; *P*=.001) compared to mid-aged generation (SMD 0.35; 95% CI −0.36 to 1.06; *P*=.34). Grouping with sample size was effective in reducing heterogeneity and the group with a larger number of patients resulted in an insignificant effect (SMD 0.16; 95% CI −0.03 to 0.35; *P*=.01). Grouping the studies by the follow-up period could also mitigate the heterogeneity, and short-term follow-up observed a more intensive effects (SMD 0.79; 95% CI 0.39-1.20; *P*<.001) in comparison to long-term follow-up (SMD 0.27; 95% CI 0.01-0.52; *P*=.04). Disease was another important source of heterogeneity, but we failed to analyze the impact of different chronic diseases on quality of life, owing to that the 6 selected studies covered 6 different chronic diseases, including atrial fibrillation, breast cancer, diabetes, heart failure, depression, and COPD.

We also found that in many studies, there were detailed data about different domains of QoL. Although scales used in these studies varied, it is still worthwhile to try and integrate the results in the shared domains if sufficient data were available (Figure 3B-F). SMD was applied to physical, mental, and social functioning, as there were no less than 2 different scales among selected studies. Two papers using EORCT QLQ-C30 contained cognitive functioning, role functioning, and MD was used to assess the data.

**Table 3:** Subgroup analysis of general quality of life score.

Subgroup and stratification		Studies, n	*P* value for heterogeneity	*I*^2^ (%)	Pooled standardized mean difference	*P* value for pooled results	References	
**Scale**								
	Disease-specified QoL^a^ scale	2	.01	84	0.62 (−0.17 to 1.42)	.12	[29,32]	
	General QoL scale	4	.002	80	0.38 (0.06 to 0.69)	.02	[15,17,21,35]	
**Age, years**								
	<55	2	.004	88	0.35 (−0.36 to 1.06)	.34	[15,17]	
	≥55	4	.03	67	0.50 (0.20 to 0.81)	.001	[21,29,32,35]	
**Sample size, n**								
	<150	3	.42	0	0.75 (0.51 to 1.00)	<.001	[15,32,35]	
	≥150	3	.17	43	0.16 (−0.03 to 0.35)	.10	[17,21,29]	
**Follow-up period, months**								
	≤2	2	.19	42	0.79 (0.39 to 1.20)	<.001	[32,35]	
	>2	4	.02	69	0.27 (0.01 to 0.52)	.04	[15,17,21,29]	

^a^QoL: quality of life.

##### Physical Functioning in QoL

Six studies reported the physical functioning domains of QoL, with a total of 866 patients involved. SMD method was used considering different measurement scales. A moderate level of heterogeneity across the studies could be observed (*P*=.09; *I*^2^=47%), so a fixed effects model was used to calculate the mean effect size. The result was that the positive effect of telehealth intervention was statistically significant (SMD 0.15; 95% CI 0.02-0.29; *P*=.03).

##### Mental Functioning in QoL

Six studies reported the mental functioning domains of QoL, with a total of 866 patients involved. SMD method was used considering different measurement scales. A moderate level of heterogeneity across the studies could be observed (*P*=.02; *I*^2^=61%), so a random effects model was used to calculate the mean effect size. The result was that the positive effect of telehealth intervention was statistically significant (SMD 0.37; 95% CI 0.13-0.60; *P*=.002).

##### Cognitive Functioning in QoL

Two studies reported the cognitive functioning domains of QoL, with a total of 415 patients involved. MD method was used as both studies used the European Organization for Research and Treatment of Cancer Core Quality of Life (EORTC QLQ-C30) questionnaire. Significant heterogeneity across the studies could be observed (*P*=.01; *I*^2^=84%), so a random effects model was used to calculate the mean effect size. The result was that the effect of telehealth intervention was not statistically significant (MD 8.31; 95% CI −7.33 to 23.95; *P*=.30).

##### Role Functioning in QoL

Two studies reported the role functioning domains of QoL, with a total of 415 patients involved. MD method was used as both studies used the EORTC QLQ-C30 questionnaire. Significant heterogeneity across the studies could be observed (*P*=.03; *I*^2^=80%), so a random effects model was used to calculate the mean effect size. The result was that the effect of telehealth intervention was not statistically significant (MD 5.30; 95% CI −7.80 to 18.39; *P*=.43).

##### Social Functioning in QoL

Three studies reported the social functioning domains of QoL, with a total of 479 patients involved. SMD method was used as Guo et al [32] used diabetes-specific QoL questionnaire [32] other than the EORTC QLQ-C30 questionnaire adopted in another 2 studies. Significant heterogeneity across the studies could be observed (*P*<.001; *I*^2^=88%), so a random effects model was used to calculate the mean effect size. A statistically significant positive effect of telehealth intervention was observed (SMD 0.64; 95% CI 0.00-1.29; *P*=.05).

#### Depression

**Figure 4:**
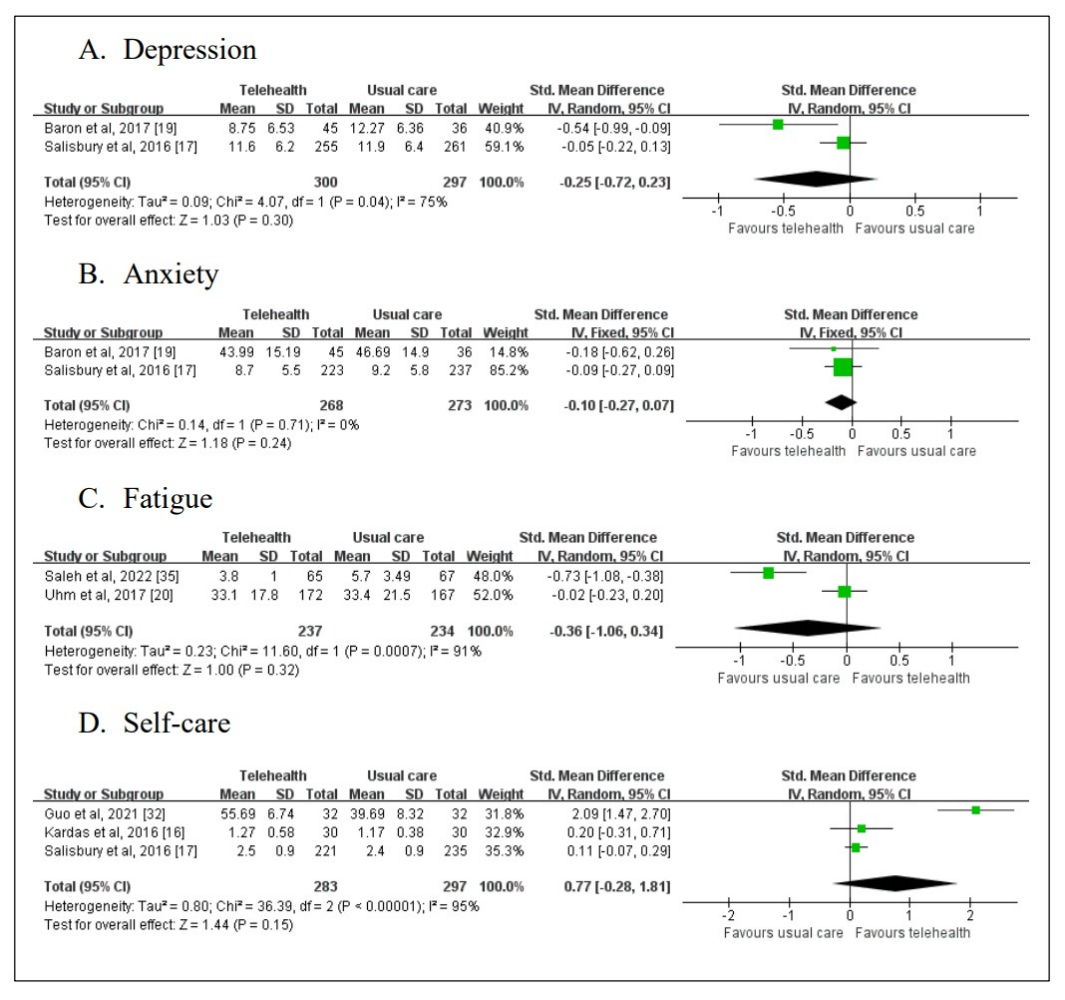
Forest plots of (A) depression, (B) anxiety, (C) fatigue, and (D) self-care.

Two studies reported depression, with a total of 597 patients involved (Figure 4A). SMD method was used considering different measurement method. Significant heterogeneity across the studies could be observed (*P*=.04; *I*^2^=75%), so a random effects model was used to calculate mean effect size. No statistically significant effect of telehealth intervention was observed (SMD −0.25; 95% CI −0.72 to 0.23; *P*=.30).

#### Anxiety

Two studies reported anxiety, with a total of 541 patients involved (Figure 4B). The SMD method was used considering different measurement methods. Homogeneity across the studies could be observed, but it was not statistically significant (*P*=.71; *I*^2^=0%). A fixed effects model was used to calculate the mean effect size. No statistically significant effect of telehealth intervention was observed (SMD −0.10; 95% CI −0.27 to 0.07; *P*=.24).

#### Fatigue

Two studies reported fatigue, with a total of 471 patients involved (Figure 4C). SMD method was used considering different measurement methods. Homogeneity across the studies could be observed, but it was not statistically significant (*P*=.71; *I*^2^=91%). A fixed effects model was used to calculate the mean effect size. No statistically significant effect of telehealth intervention was observed (SMD −0.36; 95% CI −1.06 to 0.34; *P*=.32).

#### Self-care

Three studies reported self-care, with a total of 580 patients involved (Figure 4D). SMD method was used considering different measurement methods. Significant heterogeneity across the studies could be observed (*P*<.001; *I*^2^=95%). A random effects model was used to calculate the mean effect size. No significant effect of telehealth intervention was observed (SMD 0.77; 95% CI −0.28 to 1.81; *P*=.15).

## Discussion

### Principal Results

The primary purpose of this paper is to investigate the current research status of using subjective indicators to evaluate the effectiveness of telehealth chronic management systems. The primary finding was that the frequently used subjective outcomes in this area included QoL, psychological well-being (depression, anxiety, and fatigue), medical adherence, and self-management. Subsequently, we quantitatively synthesize the effects of the telehealth chronic management system in the outcomes mentioned above.

We selected 10 high-quality RCTs with sufficient data and eligible intervention methods in the meta-analysis. The telehealth system could have a positive influence on QoL, whereas no significant effects were found on depression, anxiety, fatigue, and self-care. However, there were no more than 3 studies available for the last 4 outcomes, which might reduce the reliability of the results.

Furthermore, we carefully analyzed the effects on different subdomains in QoL, including physical, mental, social, cognitive, and role functioning. Statistically significant improvement could be observed in physical functioning and mental functioning. In these 2 areas, the results were relatively more reliable considering the number of studies involved and the heterogeneity level. We can also observe significant effects on social functioning; yet, the heterogeneity was considerable. No significant effects were found in cognitive functioning and role functioning.

Heterogeneity existed in most outcomes, except for the physical functioning of QoL and anxiety. The small number of studies and variations in patients, diseases, and telehealth systems bring considerable divergence. Subgroup analysis in QoL suggested that sample size and the duration of follow-up might be sources of heterogeneity. It is worth noting that we still observe a statistically significant improvement under moderate heterogeneity, in the physical and mental functioning of patients’ QoL. This may indicate that subjective indicators have the potential stability and applicability among multiple chronic diseases.

Although functionalities of telehealth intervention were not the focus of this study, we could still derive some observations from pairwise comparison. Two experiments conducted by Galiano-Castillo et al [15] and Uhm et al [20], respectively, in 2017, tested the effectiveness of remote exercise programs among breast cancer survivors, using the same QoL scale. However, the former study yielded better results in 5 domains of QoL. Comparing these 2 studies' characteristics, the major differences are age and individual supervisors. On average, patients in Galiano-Castillo’s [15] group were 11.3 years younger, and each individual received instant supervision from the research staff. While the effects of age might be insignificant based on the subgroup analysis, close monitoring and guidance might be a potential contributor to the effectiveness of TCDMS that was worth further study.

### Advantages

Inconsistency among studies results was a critical issue in such a field. In this study, we struck a balance between managing the heterogeneity among selected studies and assessing subjective indicators’ applicability to various chronic diseases. We set some restrictions on publish time and intervention design, hoping to synthesize the latest outcomes with eligible telehealth systems. The telehealth systems were required to at least have telemonitoring and management functions, but each study may have different focuses, such as on exercise or disease monitoring. In contrast, platforms with mere health education functions were excluded. In this scenario, we could analyze the applicability of subjective indicators to various chronic diseases, including cardiovascular diseases, cancer, diabetes, COPD, depression, and hypertension.

### Limitations and Future Research

The main limitation is the limited number of research included in the meta-analysis, and consequently, the synthesized results were less robust. Despite a surge in the number of research on telehealth since 2015, the strictly designed RCTs for evaluating the system’s effectiveness are still lacking. In particular, there is a shortage of studies that evaluated beyond clinical outcomes to subjective outcomes such as QoL or psychological well-being. Additionally, few studies experimented TCDMS’s effects among multiple chronic disease groups. Thus, this paper can only evaluate the effectiveness by combining the results of different studies on different chronically ill groups.

Other limitations during the meta-analysis process include (1) although this study strictly followed the Preferred Reporting Items for Systematic Reviews and Meta-Analyses statement, there might also be subjective bias in study selection and risk analysis. (2) We failed to access the full text of nearly a hundred papers, affecting the number of papers included. (3) Participants mainly concentrated on those using the eHealth platform, which might not apply to the elderly and low education group.

This study only listed indicators that had been used in selected studies. Most of the selected indicators assess the effectiveness from a patient perspective, but subjective indicators could also be extended to social level, such as cost-effectiveness, social acceptance, and so forth. More research can study the effects of TCDMS on other parties involved, such as caregivers, the community, the hospital, and the government.

In the future, we hope a systematic evaluation system could be developed with the joint efforts from the experts in the fields of chronic disease control, community management, and technology. Evaluation matters, as it leads the industry development, but at present, the changing page of assessment does not match with the pace of technology changes in chronic health care. Therefore, with the ongoing trend of long-term daily health monitoring and management, we expect more research on the comprehensive assessment system on the effectiveness of telehealth.

### Conclusions

This review summarized the subjective indicators used to estimate the effectiveness of the TCDMS. Common indicators included QoL, psychological well-being (depression, anxiety, and fatigue), medical adherence, and self-management. Meta-analysis showed that the current telehealth system could improve the QoL, especially in the physical, mental, and social functioning domains. However, we did not obtain statistically significant results in the cognitive and role functioning of the QoL. Moreover, no significant effects were observed in psychological well-being and self-management score. Limited number of selected studies mainly constricted this study. Further experiments and research were warranted to integrate subjective outcomes into evaluation systems of the TCDMS.

## Appendix

Multimedia Appendix 1 Table of characteristics of selected studies.

## Abbreviations

COPD: chronic obstructive pulmonary disease
EORTC QLQ-C30: European Organization for Research and Treatment of Cancer Core Quality of Life
MD: mean difference
QoL: quality of life
RCT: randomized controlled trial
SMD: standardized mean difference
TCDMS: telehealth chronic disease management system
