# Supplementary material for: Evaluation of the Effectiveness of Telehealth Chronic Disease Management System: Systematic Review and Meta-analysis
Source: J Med Internet Res. 2023 Apr 27;25:e44256. doi: 10.2196/44256 (PMC10176143; doi:10.2196/44256)
Supplement: Multimedia Appendix 1 [file jmir_v25i1e44256_app1.docx]

| Study | Country | Included in meta analysis? | Participants | | | | | Follow-up | Intervention | | Outcomes |
| --- | --- | --- | --- | --- | --- | --- | --- | --- | --- | --- | --- |
|  |  |  | Recruitment | Sample  , I:C | Total | Sex,  M:F | Age,  yr  (SD) |  | Int. | Con. |  |
| Galiano-Castillo ,  2016[15] | Spain | Yes | Breast cancer survivor from Oncology and Breast units at the Virgen de las Nieves Hospital (Granada, Spain). | 40:41 | 81 | - | 48.3  (8.8) | 8 weeks & 6 months | e-CUIDATE system that facilitates remote rehabilitation by offering exercise program and enabling self-monitoring. Patient’s records are processed remotely and research staff would call patients regularly | Receive basic recommendations (written format) for exercise | QoL (EORTC QLQ-C30)  Pain (BPI)  Fatigue (R-PFS)  Adherence rate (attendance rate) |
| Kardas, 2016[16] | Poland | Yes | 18-65 years old patient diagnosed with type 2 diabetes at least 6 months before study | 30:30 | 60 | 36:24 | 59.5  (6.9) | 6 weeks | COMMODITY12 system composed of a smart phone and wirelessly connected sensors that enables medical data analysis | Usual care | HRQOL(EuroQol-5D-5L)  Adherence  (Dose taken) |
| Salisbury, 2016[17] | U.K. | Yes | Patients with depression, aged 18 years or older | 307:302 | 609 | 192:417 | 49.5  (12.8) | 4 months & 8 months & 12 months | Patients received usual care plus intervention, which included a Healthlines web portal, linked to medical resources, depression monitoring modules and forum. | Usual care | Anxiety (GAD-7)  Depression  (PHQ-9)  QOL(EQ-5D-5L)  Self-efficacy(HeiQ) |
| Baron, 2017  [18,19] | U.K. | Yes | 18 years old and above patients with poorly controlled type1 or type 2 diabetes | 45:36 | 81 | 46:35 | 57.1  (13.6) | 3 months & 9 months | MTH equipment, mobile-phone software and relevant training that facilitate self-monitoring and telemonitoring | Follow-up appointment with diabetes specialist nurse every 3 to 4 months | Depression  (CESD-10)  Anxiety (STAI-6)  HRQOL(SF-12)  Self-efficacy (HeiQ)  Self-care  (SDSCA) |
| Uhm, 2017  [20] | Korea | Yes | Patients that were histologically confirmed breast cancer, age between 20 and 70 years | 179:177 | 356 | - | 50.3  (9.5) | 6 weeks & 12 weeks | mHealth with pedometer provided with a smartphone exercise application to encourage patients to exercise | Receive an exercise brochure | HRQOL(EORTC-QLQ-C30)  physical activity (IPAQ-SF) |
| Tupper, 2018[21] | Denmark | Yes | Patients with severe and very severe, stable COPD at high risk of exacerbations and hospital admissions | 141:140 | 281 | 137:144 | 69.6  (9.6) | 6 months | Patients reported clinical condition through tablet computer. The system offered remote measurement every 4 weeks and consultations without video | Scheduled visits at the outpatient clinics once or twice a year | HRQOL(15D QOL questionnaire) |
| Ren, 2019[22] | China | No | Patient with hypertension (blood pressure over 130/85 mmHg or taking antihypertensives) or diabetes (FBG over 5.55 mmol/L or taking anti-diabetes drug), age over 40 years old | 139:142 | 281 | 116:165 | 59.4  (10.0) | 6 months | Install a mobile health app and receive a smart band to monitoring everyday physical and clinical condition. Specialists gave advice based on recorded data. | Communities offered regular diabetes management services. Specialist offers regular medical suggestions and prescription | Medical Adherence (self-reported adherence)  Physical Activity (GPAQ) |
| Zhao, 2019[23] | China | No | Patients diagnosed with type 2 diabetes, age between 40 and 80 | 37:38 | 75 | 49:26 | 58.3  (8.5) | 3 months | Participating in diabetes-related lectures and using a mobile health App that has three main functions 1) notification and message; 2) index measurement 3) data analysis | Participating in diabetes-related lectures only | Self-efficacy  (KBA)  Self-management  (KBA)  Social support  (PROMIS) |
| Wagenaar, 2019[24] | Netherlands | No | Patients with heart failure, age over 18 | 150/150 | 300 | 222:78 | 66.8  (11.1) | 3 months & 6 months & 9 months | Patient used e-Vita platform to record clinical data, automatically analyse the data and nurses contact patient if there is any abnormal condition. | Receive usual care from heart failure outpatient team, which consisted of on average 4 routine consultations a year | HRQOL (Minnesota living with HF questionnaire) Self-care (EHRScB) |
| Schoenthaler, 2020[25] | U.S. | No | Patients who self-identified as African American, received care at the primary care clinic, had uncontrolled hypertension, at least 18 years old | 21:21 | 42 | 23:19 | 57.6  (5.6) | 3 months | Receive mHealth intervention consisted of 3 main parts: 1) a tailoring survey of adherence questionnaire 2) an individualized adherence-promoting adherence profile 3) a personalized list of interactive adherence-promoting modules | Complete the introductory tailoring survey on the same platform; but did not receive the results display their adherence barriers | Adherence  (MMAS-8)  Health literacy  (s-TOFHLA) |
| Broers, 2019[26] | Spain & Netherlands | No | Patient primarily diagnosed with cardiovascular disease, age between 18 to 75 years | 76:74 | 150 | 107:43 | 62.0  (11.6) | 3 months & 6 months | Receive technological devices, including Fitbit smart watch, Beddit sleep tracker, etc., to obtain objective measures on patients’ physical functioning | Did not receive wearable and monitoring devices, only receive the treatment as usual | Depression  (PHQ-9)  Anxiety (GAD-7) QOL(WHOQOL)  Life-style (HPLP)  Satisfaction  (CSQ-8)  Type D Personality  (Type D scale) |
| Cichosz, 2018[27] | Denmark | Yes | Patients with heart failure and were placed within the New York Heart Association class 2, 3, or 4. | 93:100 | 193 | 141:52 | 70.5  (-)^a^ | 12 months | Patient was engaged in their own illness through self-monitoring. Specially trained nurses assessed all the data in system and patients were contacted if data were not received as planned. | Receive usual care only that consisted of usual treatment, monitoring and care | HRQOL(SF36) |
| Clark, 2020[28] | U.S. | No | Patients diagnosed with type 2 diabetes and poor glycemic control Age between 18 and 75 years | 63:63 | 126 | 32:94 | 48.4  (9.8) | 3 months & 6 months | Usual care and receive motivational, educational or call to action text message over a 6-month period every 2 to 3 per day, and was encouraged to text message back. | Usual care including visits with a primary care physician, certified diabetes educator and group DSME/S | Distress (DDS) |
| Caceres, 2020[29] | U.S. | Yes | Patients over the age of 18 with documented atrial fibrillation (AF) and under AF treatment | 115:123 | 238 | 183:55 | 61.3  (11.8) | 6 months | iHEART intervention, which consisted of an iPhone equipped with a mobile ECG system to records clinical conditions. Patients will receive provider caring once abnormal clinical situation was captured | Receive usual care | HRQoL (AFEQT, SF36, EQ-5D)  QALYs (EQ-5D index) |
| Van der Hout, 2020[30] | Netherlands | No | Patients were cancer survivors, age over 18 years and 3 months to 5 years after treatment | 320:305 | 625 | 316:309 | 65  (-)^a^ | 3 months & 6 months | Use Oncokompas which supports self-management in cancer survivors, by monitoring symptoms and health related quality of life | Receive usual care and will have access to Oncokompas after 6 months | QALYs (EQ-5D) |
| Clays, 2021[31] | Belgium & Italy | No | Adult ambulatory congestive heart failure patient in a stable condition | 34/22 | 56 | 43:13 | 63.1  (10.5) | - | Patients in the intervention group continued to receive usual care, and additionally used the HeartMan personal health system in their home setting | Receive usual care | Depression (BDI)  Anxiety (STAI-Y)  HRQOL(MLHFQ) Self-care (SCHFI) |
| Guo, 2021[32] | China | Yes | Patients diagnosed with type 2 diabetes, age between 18 and 75 years | 32:32 | 64 | 39:25 | 57.3  (14.4) | 4 weeks | Receive mHealth management based on the mHealth management model that consisted of a network platform, a implantable glucose sensor, a mobile app and GP support | Receive usual health management. GP conducted telephone follow-ups once a week. Health education material is delivered at follow-up visit | HRQOL (diabetes specific QOL) Self-care(SDSCA) |
| Zhang, 2021[33] | China | No | Patient suffering from pain that met cancer pain diagnostic criteria, age over 18 years | 51:49 | 100 | 72:28 | 56.6  (14.5) | 4 weeks | Patient received daily 24-hour pain diaries, ADR forms every 3 days and BPI form every 15 days | Receive conventional care and detailed medication education | Adherence (MMAS)  Pain (BPI) |
| Vos, 2021[34] | Netherlands | No | Patients receiving primary surgical treatment for stage I-III colon cancer or rectosigmoid carcinoma and qualified for routine follow-up | 68:73  83:79 | 141  162 | 98:43  105:57 | 68.0^a^  68.5^a^ | 3 months & 6 months & 12 months | Divided into 2 subgroup controlled trial:   1. General-practitioner-led care group 2. Surgeon-led care group   Patient uses Oncokompas that enables self-management | 1)General-practitioner-led care without Oncokompas access 2)Surgeon-led care group without Oncokompas access | HRQOL (EORTC QLQ-C30) |
| Saleh, 2022[35] | Jordan | Yes | Patients with heart failure | 65:67 | 132 | 80:52 | 60.8  (10.5) | 8 weeks | Use a mobile health app tailored to decrease the time spent in sedentary behaviours and to increase the time spent in physical activities performed at light and creator intensity levels | Medical management by information guidance offered by a cardiologist for patients with Heart Disease | HRQOL (SF-36),  Symptom burden (MSAS-HF) |

^a^ These studies do not provide mean and standard deviation for age. Instead, they provide median, 25^th^ and 75^th^ percentile. We use the median to approximate the mean age and omit standard deviation.
